# Long short-term memory models of water quality in inland water environments

**DOI:** 10.1016/j.wroa.2023.100207

**Published:** 2023-11-16

**Authors:** JongCheol Pyo, Yakov Pachepsky, Soobin Kim, Ather Abbas, Minjeong Kim, Yong Sung Kwon, Mayzonee Ligaray, Kyung Hwa Cho

**Affiliations:** a Department for Environmental Engineering, Pusan National University, Busan 46241, Republic of Korea; b Environmental Microbial and Food Safety Laboratory, USDA-ARS, Beltsville, MD, USA; c School of Civil, Urban, Earth, and Environmental Engineering, Ulsan National Institute of Science and Technology, 50 UNIST-gil, Ulju-gun, Ulsan 44919, Republic of Korea; d Physical Sciences and Engineering, King Abdullah University of Science and Technology, Thuwal 23955-6900, Kingdom of Saudi Arabia; e Disposal Safety Evaluation R&D Division, Korea Atomic Energy Research Institute (KAERI), 111, Daedeok-daero 989 beon-gil, Yuseong-gu, Daejeon 34057, Republic of Korea; f Environmental Impact Assessment Team, Division of Ecological Assessment Research, National Institute of Ecology, Seocheon, Republic of Korea; g Institute of Environmental Science and Meteorology, College of Science, University of the Philippines Diliman, Quezon City 1101, Philippines; h School of Civil, Environmental and Architectural Engineering, Korea University, Seoul 02841, Republic of Korea

**Keywords:** Long short-term memory, Inland water, Water quality, Ensemble LSTM, Deep learning models

## Abstract

Water quality is substantially influenced by a multitude of dynamic and interrelated variables, including climate conditions, landuse and seasonal changes. Deep learning models have demonstrated predictive power of water quality due to the superior ability to automatically learn complex patterns and relationships from variables. Long short-term memory (LSTM), one of deep learning models for water quality prediction, is a type of recurrent neural network that can account for longer-term traits of time-dependent data. It is the most widely applied network used to predict the time series of water quality variables. First, we reviewed applications of a standalone LSTM and discussed its calculation time, prediction accuracy, and good robustness with process-driven numerical models and the other machine learning. This review was expanded into the LSTM model with data pre-processing techniques, including the Complete Ensemble Empirical Mode Decomposition with Adaptive Noise method and Synchrosqueezed Wavelet Transform. The review then focused on the coupling of LSTM with a convolutional neural network, attention network, and transfer learning. The coupled networks demonstrated their performance over the standalone LSTM model. We also emphasized the influence of the static variables in the model and used the transformation method on the dataset. Outlook and further challenges were addressed. The outlook for research and application of LSTM in hydrology concludes the review.

## Highlights

• This study reviewed LSTM applications of water quality prediction of inland water.
• LSTM models demonstrate better performance for water quality prediction.
• LSTM can be further enhanced by combining it with other machine learning approaches.
• Non-time series-type data present challenges and opportunities for LSTM prediction.

## 1 Introduction

Water monitoring and management of water quality have provided diverse and numerous datasets that have been facilitated by the realization of the impact of global climate change on water resources and by advances in sensor technology (Weber et al., 2018). The efficient use of an immense amount of data yields enormous benefits when complex systems, such as river and lake systems, are analyzed (Christ et al., 2016). However, water quality prediction with those numerous datasets is still challenging due to multitude of dynamic and interrelated factors, including climate conditions, landuse and seasonal changes. There have been many efforts for developing numerical water quality models such as open-source model of high transferability (Delft3D), Environmental Fluid Dynamics Code (EFDC), and Water Quality Analysis Simulation Program (WASP), but the complexity and uncertainty associated with accurately representing the multitude of processes that influence water quality have been always issued (Beck, 1987; Lindenschmidt, 2006; Xu et al., 2022a,b). Recently, deep learning modeling has become a fruitful approach that has been widely used in water quality modeling. Machine learning models find, or extract features, that is, independent combinations of measurable variables that serve as better predictors or classifiers. These features provide a high-level representation of complex datasets and relationships within them. Such models have been successfully used to estimate various water quality variables including total nitrogen (Sha et al., 2021), total phosphorus (Zhang et al., 2020a), dissolved oxygen (Moghadam et al., 2021), chlorophyll-a (Guo et al., 2022), total organic carbon (Zhu et al., 2019), turbidity (Wan et al., 2022), and colored dissolved organic matter (Niu et al., 2021).

In the original applications of machine learning to predictions of environmental time series such as water quality and metrological data, cells of artificial neural networks (ANNs) having parameters that needed to be determined to provide the best fit used sections of fixed length as inputs. Eventually, it was found that substantially better predictions in time series could be provided with recurrent neural networks (RNNs). A cell of RNNs uses not only current datasets-predictors as inputs but also the output of the cell obtained after its application to the previous input dataset. Thus, previous information is better connected to the present prediction task (Olah, 2015). In applications to time series, RNNs use data sequences of fixed length as the dataset-predictors and can simulate the effect of distant past events on current data in theory. However, in practice, training the RNN, that is, changing its parameters to fit the data, may be stalled because of the unavoidably limited accuracy of the computations. For the same reason, the parameter-search process may vary. These phenomena are known as vanishing gradient and exploding gradient problems. These problems have been alleviated by the introduction of Long Short-Term Memory (LSTM) neural networks (Sherstinsky, 2020). LSTM models are not sensitive to the fact that the time interval between the current observation and the event affecting this observation is unknown and can be large. This capability of LSTM is caused by utilizing the cell state variables that have not existed in other ANN architectures and has allowed selective remembering and forgetting of the sequential information of data (Xu et al., 2022a,b). LSTM is the most cited neural network architecture introduced in the 20th century (Schmidhuber, 2021).

Several studies have been conducted to predict the quality of inland water by applying the LSTM model (e.g., Hu et al., 2019; Lee and Lee, 2018; Liu et al., 2019; Wang et al., 2017). Water quality modeling has expanded to multi-dimensional data, such as multi-spectral imagery, radar images, and GIS-based data (Srivastava et al., 2022). Sagan et al. (2020) successfully applied LSTM model with reflectance spectra data of Landsat-8 and Sentinel-2 to estimate water quality variables such as cyanobacteria, algae, total suspended solid, turbidity, nitrate nitrogen, phosphate phosphorus, and total dissolved solid. In addition, it was found that LSTM performance could be improved by combining LSTM with different deep learning techniques. In particular, the increasing complexity of water-quality-related data sequences has led to the need for feature extraction improvements (Fan et al., 2022; Hao et al., 2022). One of the successful methods of feature extraction is implemented in convolutional neural networks (CNNs) (Wen et al., 2017). The combinations of LSTM and CNNs have been applied in many research fields, demonstrating performance improvement compared with the use of LSTM alone (Kim and Cho, 2019; Vidal and Kristjanpoller, 2020; Zhao et al., 2019). Transfer learning is another method used to build robust deep learning models by extracting data features more flexibly (Abubakr et al., 2022). The weights of the previously developed model were used as reliable initial values for training with similar datasets (Wahlang et al., 2022). The flexibility and robustness of the transfer learning of LSTM have been proven in several studies (Bashar et al., 2020; Jung et al., 2019; Ma et al., 2020a,b). Moreover, attention networks have been applied to improve the feature extraction of deep learning models (Xie et al., 2022). The selective refinement of the learnable parameters-weights in the attention mechanism emphasizes the important features of the data, contributing to the improvement of the LSTM model performance (Kumar et al., 2020; Shen et al., 2020; Zhang et al., 2020a).

This paper reviews applications of the LSTM model for water quality modeling of inland waters. We review the opportunities of LSTM performance enhancement in water quality applications, identify promising avenues for further research, and indicate possible advances in water quality management using LSTM-based techniques.

## 2 LSTM for water quality modeling

### 2.1 LSTM theory

**Fig. 1:**
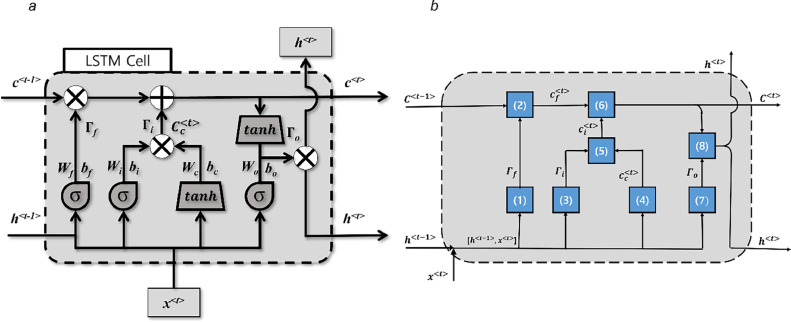
Architecture of LSTM cell (a) Information flow according to the LSTM model Eqs. (1)–(6). Numbers of equations are in parentheses. Explanation of symbols is in the text following the equations. (b) The commonly used representation of the LSTM cell explicitly showing the nonlinear transformations within the LSTM cell.

To make the LSTM model capable of learning long-term dependencies in a time series, the LSTM cell has a more complex design than that of conventional ANNs or RNNs (Lim and Zohren, 2021; Sezer et al., 2020). Specifically, the LSTM cell has an internal state variable, which, along with the LSTM output from the previous time step, is used as the input for this cell at the current time step. The equations controlling the information flow in the LSTM cell are given below, and complementary Fig. 1(a) and (b) illustrates.

(1) $$\Gamma_{f}=\sigma \left(W_{f}\left[h^{\langle t-1\rangle },x^{\langle t\rangle }\right]+b_{f}\right),$$

(2) $$c_{f}^{\langle t\rangle }=\Gamma_{f}⊙C^{\langle t-1\rangle }$$

(3) $$\Gamma_{i}=\sigma \left(W_{i}\left[h^{\langle t-1\rangle },x^{\langle t\rangle }\right]+b_{i}\right),$$

(4) $$c_{c}^{\langle t\rangle }=tanh\left(W_{c}\left[\;h^{\langle t-1\rangle },x^{\langle t\rangle }\right]+\;b_{c}\right),$$

(5) $$c_{i}^{\langle t\rangle }=\Gamma_{i}⊙\;c_{c}^{\langle t\rangle }$$

(6) $$C^{\langle t\rangle }=c_{f}^{\langle t\rangle }\;+\;c_{i}^{\langle t\rangle },$$

(7) $$\Gamma_{o}=\sigma \left(W_{o}\left[h^{\langle t-1\rangle },x^{\langle t\rangle }\right]+b_{o}\right),$$

(8) $$h^{<t>}=\Gamma_{o}\;⊙\;tanh\;c^{<t>}$$

where C indicates the cell state variable, *h* be the hidden state variable (also known as the cell output variable), and *x* be the input dataset. All input and output variables $h^{\langle t-1\rangle }$, $C^{\langle t-1\rangle }$, $x^{\langle t\rangle },\;C^{\langle t\rangle }$, and $h^{\langle t\rangle }$as well as the intermediate variables $c_{f}^{\langle t\rangle }$, $c_{c}^{\langle t\rangle }$, and $c_{i}^{\langle t\rangle }$ are vectors. The LSTM cell includes operators called ‘gates’ that modify the information as it moves through the cell. Eqs. (2), (5), and (8) describe the work of the forget, input, and output gates denoting to $\Gamma_{f}$, $\Gamma_{i}$, and $\Gamma_{o}$ respectively. The arguments of the sigmoidal functions include the vectors of biases $b_{f}$, $b_{i}$, or $b_{o}$, and the products of matrices of weights $W_{f}$, $W_{i}$, and $W_{o}$ and the vector $\left[h^{\langle t-1\rangle },x^{\langle t\rangle }\right]$, which is the concatenation of the vector of hidden state values $h^{\langle t-1\rangle }$ and input vector $x^{\langle t\rangle }$. The symbol ⊙ denotes the Hadamard product of the two vectors when elements corresponding to the same row are multiplied. At each step, an LSTM cell receives $x^{\langle t\rangle }$ as inputs and produces $C^{\langle t\rangle }$ and $h^{\langle t\rangle }$ as outputs.

### 2.2 Water quality simulation of LSTM and process-driven numerical models

A wide range of process-driven modeling tools are available which can simulate spatial and temporal evolution of water quality parameters in surface waters. These include Hydrological Simulation Program FORTRAN (HSPF) (Bicknell et al., 1997), WASP (Wool et al., 2020), Integrated Catchment (INCA) (Whitehead et al., 2016), enhanced stream water quality model (QUAL2E) (Brown and Barnwell, 1987), one-dimensional river and stream water quality model (QUAL2K), one-dimensional river model (MIKE11) (Havnø et al., 1995), EFDC (Hamrick, 1992) and Delft3D (Roelvink and Van Banning, 1995) among others. These are time-proven models which have been used in hundreds of water quality simulation studies worldwide (Tong et al., 2022). To simulate fate and transport of conservative substances, these models incorporate modeling of advection and dispersion phenomena. For non-conservative pollutants, both chemical and biological transformations are taken into consideration. While models like HSPF, INCA, and Soil and Water Assessment Tool (SWAT) are suitable for conducting 1D simulations of surface water quality constituents at the catchment scale, more detailed and comprehensive simulation outcomes can be achieved through the use of numerical models such as WASP, Delft3D, and EFDC (Bouraoui and Grizzetti, 2014; Keller et al., 2023; Zhou et al., 2023).

One advantage of numerical water quality models over LSTM is that they can provide detailed 3-dimensional fate and transport of pollutants in geometrically complex water bodies (Cho et al., 2020). This detailed simulation can provide profound insights into the behavior of pollutants in aquatic environments. However, these high-resolution simulations come at the cost of computation resources. Furthermore, these numerical models leverage mass conservation equations which forces these models to follow laws of nature during simulation (Abbaspour et al., 2015). The ability to simulate detailed processes in complex environments while following physical laws increases representative capacity of these numerical models (Clark et al., 2015). However, the effectiveness of numerical models because of their high representation of underlying processes depends on discretization step used to determine the grid. While a smaller grid size can simulate detailed processes however it exponentially increases the computation demand (Leng et al., 2022). On the other hand, larger grid size can omit important details thereby compromising model's efficacy. Despite these advantages, one limiting factor in the application of these numerical models is their requirement for detailed input information. This information is often required in the form of boundary or initial conditions which are often difficult to measure. Another limitation of these models is that they cannot incorporate a wide variety of input data. On the other hand, LSTM based deep learning models can process numerical, image, or text information simultaneously (Ward et al., 2020). This flexibility of LSTM has the potential to build more representative water quality models by incorporating wide range of factors affecting the target variable (Brunner et al., 2021).

Another advantage of numerical models over LSTM based deep learning models is the interpretation of parameters of calibrated models (Tsai et al., 2021). The parameters of a well calibrated numerical water quality model reveal important insights about the physical world (Feng et al., 2022). For example, parameters K12C, K12T and KNIT in WASP are related to nitrification while CCHL, HNRP and KMNG correspond to nutrient update by algae (Wool et al., 2020). Similarly, parameters KT and BM of EFDC model, describe effect of temperature and basal metabolism on cyanobacteria (Hamrick, 1992). However, a similar interpretation of an LSTM model with high prediction performance is not possible. Research has been carried out to interpret either the parameters of trained LSTM or intermediate outputs from a trained LSTM model. Kratzert et al. (2019a,b) showed correspondence between hydrological system (snow-water equivalent and soil moisture) and LSTM behavior. A similar interpretation of a trained LSTM for water quality parameters can also be performed in future.

### 2.3 Performance of LSTM compared with other machine learning models

LSTM models have demonstrated performance in water quality prediction than other machine learning models by showing higher correlation coefficient and lower error. AlDahoul et al. (2021) conducted experiments to evaluate LSTM's performance compared to other models. The first trial predicted sediment concentration for the next day using 10 years of data. Four models—ElasticNet LR, Multilayer Perceptron Neural Network, Extreme Gradient Boosting, and LSTM—were tested, with LSTM consistently outperforming others. LSTM also competed with ElasticNet LR in predicting monthly sediment levels despite a smaller dataset. In the third experiment, LSTM excelled in forecasting sediment concentrations seven days ahead. Overall, LSTM's predictive power exceeded ElasticNet LR's across scenarios. Dong et al. (2021) showed LSTM's superiority over traditional neural networks in predicting windspeed data collected every five minutes over a year. LSTM's success stemmed from capturing nonlinear features in data sequences, unlike traditional neural networks (Lagesse et al., 2020; Li et al., 2017). Shewalkar (2019) highlighted LSTM's advantages in speech recognition over various methods, citing its computational efficiency, high accuracy, and robustness. In essence, the collective evidence highlights LSTM's dominant performance across various domains and challenges, underscoring its potential as a powerful tool in predictive modeling, particularly for time-series and sequence-dependent data.

### 2.4 LSTM enhanced by preprocessing

Environmental data have static and dynamic variables with different kinds and different time scales. Thus, Data preprocessing is crucial for enhancing the performance of LSTM models due to noise reduction, feature scaling, and improving convergence. Recent studies have shown that preprocessing the original data prior to training yields improved LSTM results (Sha et al., 2021; Sun and Huang, 2020). In a study conducted by Sha et al. (2021), they employed the Complete Ensemble Empirical Mode Decomposition with Adaptive Noise (CEEMDAN) method to separate noise and periodic components from data. These extracted components were used as inputs for the LSTM model, resulting in improved model performance. However, the application of CEEMDAN had differing effects on non-periodic (total nitrogen, TN) and strong-periodic (dissolved oxygen, DO) water quality parameters, with TN improvements being significantly greater than those for DO.

Dong and Zhang (2021) introduced a two-stage decomposition technique involving CEEMDAN and variational mode decomposition (VMD) to analyze polycyclic aromatic hydrocarbon concentration time series. The LSTM was then applied to explore the characteristics of each subsequence, leading to enhanced prediction performance. Notably, the additional application of VMD further improved predictive capabilities. Despite CEEMDAN's efficiency with complex and variable data, room for improvement in predictive performance remains due to inadequately extracting high-frequency features from signals. Hence, the CEEMDAN–VMD two-stage decomposition approach maximizes information from the PAH time series and significantly enhances model prediction performance (Li et al., 2021). Wang et al. (2023) introduced a short-term water quality prediction model designed to address the nonlinear, unstable, and random nature of water quality parameters. This model combines VMD to enhance LSTM performance. Initially, VMD decomposes water quality data into stable components, reducing data instability and improving predictability. Each component is then used in the LSTM model for prediction.

Nourani and Behfar (2021) introduced two LSTM variants, namely SLSTM and WLSTM, for estimating suspended sediment load (SSL) at gauging stations in the Missouri and Upper Mississippi areas. SLSTM captures time-series seasonality of the elements. WLSTM employs wavelet transform to decompose signals into distinct patterns for input. Both models effectively identify SSL's long-term properties, enhancing performance (Foufoula-Georgiou et al., 1994).

The Synchrosqueezed Wavelet Transform (SWT, Daubechies et al., 2011) enhances time-frequency resolution of non-stationary signals by reorganizing the continuous wavelet transform's time-frequency map along the frequency axis. SWT also exhibits robustness against noise (Herrera et al., 2014). Song et al. (2021) integrated LSTM with SWT to denoise water quality data and enhance model performance. Using 365 weekly dissolved oxygen (DO) values from two stations in the Haihe River Basin, they showcased improved outcomes through the noise-resistant capabilities of SWT and LSTM's nonlinear mapping. A limitation was the insufficient data to fully explore all of SWT's advantages.

## 3 LSTM combination with CNN

LSTM networks have been pivotal in modeling time-series water quality due to their ability to simulate long-range dependences, but have limitations when applied standalone to water quality prediction. In particular, using LSTMs on multiple-dimensional data of water quality can result in inefficient resource usage. Barzegar et al. (2020) used the low performance of LSTM standalone model to predict DO and Chl-a in the Small Prespa Lake in Greece. This is because combining LSTM and CNN can provide significant improvement of model accuracies by leveraging complementary data.

### 3.1 LSTM-CNN theory

A hybrid model combining CNN and LSTM improves feature learning from complex inputs. A CNN is a feedforward neural network that uses deep convolutional operations to enhance feature extraction (Kim and Cho, 2019; Yan et al., 2021). A typical CNN structure includes a convolutional layer, pooling layer, fully connected layer, and an output layer. The convolutional layer performs computations with learnable weights and biases in the convolutional filters for input transformation and feature extraction (Eq. (9)). The pooling layer conducts downsampling of convolutional features. This can reduce the training cost of the model owing to the lower number of parameters, and the max-pooling layer extracts the maximum values from the convolutional features (Eq.10). Finally, the fully connected layer integrates the feature information by flattening the local feature maps into a one-dimensional feature vector, and then delineates it to the output layer (Eq. (11)).

(9) $$x^{l}=f_{a}\left(\sum w^{l}⊙x^{l-1}+b^{l}\right)$$

(10) $$x_{ij}^{l}=Max_{k=0,..n,s=0,..n}\left(x_{\left(i+k\right)\left(j+s\right)}^{l}\right)$$

(11) $$y=f_{a}\left(wx_{flat}^{l}+b\right)$$

where $x^{l}$ is the convolutional operation output in the lth layer, $x^{l-1}$ is the convolutional output in the *l*-1th layer, $f_{a}$ is the activation function, $w^{l}$ is the learnable weight in the lth layer, $b^{l}$ is the bias in the *l*th layer, Max indicates the max-pooling operator, $x_{ij}^{l}$ is the maximum output in elements *I* and *j* in the lth layer, $x_{flat}^{l}$ is the flattened output of the lth convolutional layer, and *y* is the output.

**Fig. 2:**
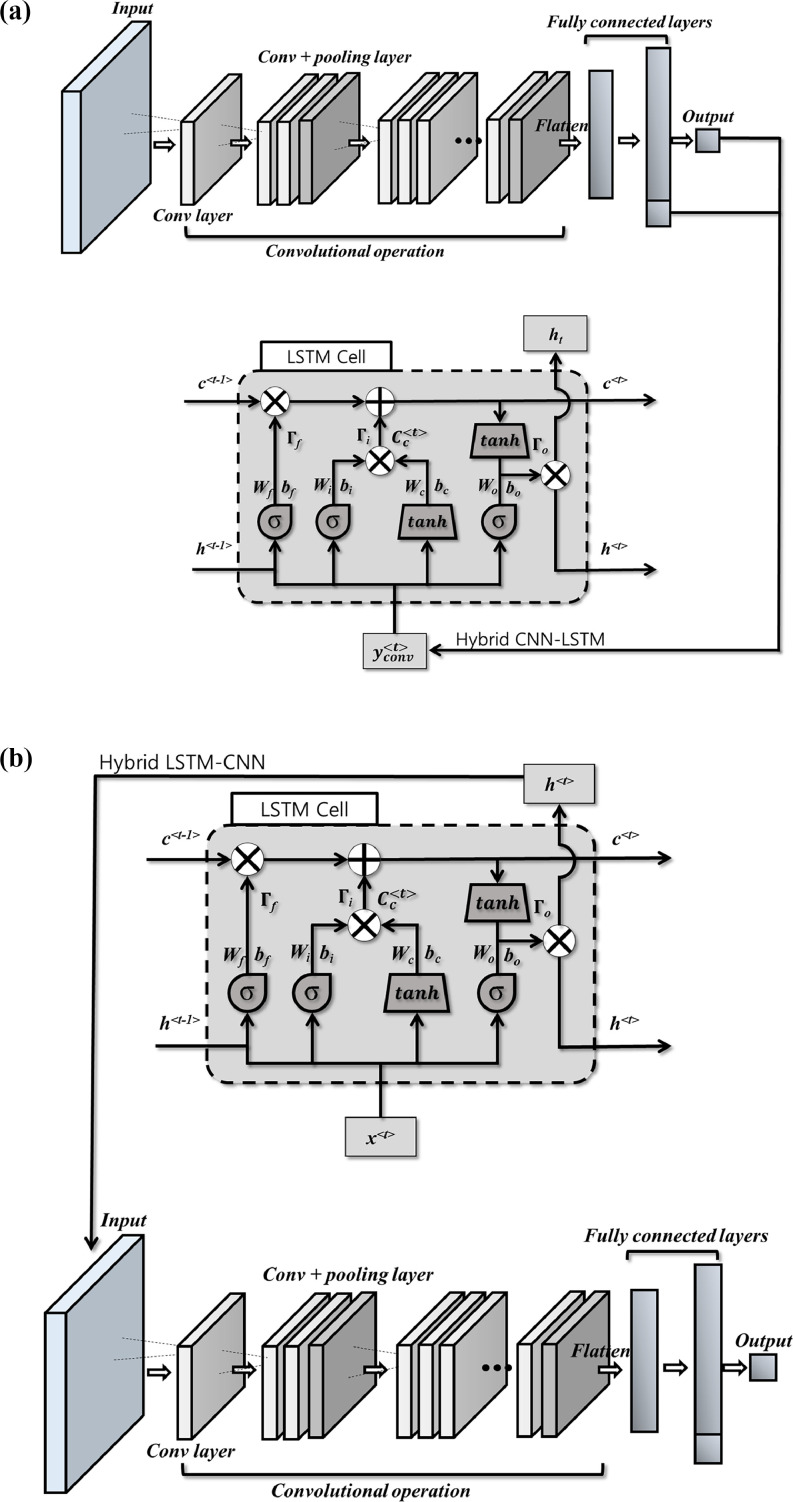
Combination of the LSTM and CNN networks to hybrid models (a) CNN-LSTM and (b) LSTM-CNN.

In Fig. 2(b), the nodes of the fully connected neurons from the CNN model are fed to the LSTM cell, as shown in Fig. 1. The vector of the CNN outputs $y_{\mathrm{conv}}^{\langle t\rangle }$ plays the role of the vector $x^{\langle t\rangle }$ in Fig. 1. The way of combining the CNN and LSTM models can vary depending on the research purpose, input dimensionality, and feature extraction priority. One representative hybrid network is the LSTM-CNN model (Fig. 2(b)). In this network structure, LSTM extracts time-series information of the input data, and then the periodic features are used as the input of the consecutive CNN model training for classification or regression tasks. LSTM cell output $h^{\langle t\rangle }$serves as the input for the CNN.

### 3.2 LSTM-CNN application for water quality prediction

The CNN-LSTM experiment predicting water quality was conducted by Baek et al. (2020) in the Nakdong River basin, simulating TN, total phosphorus, and total organic carbon levels. Using radar images, temperature, evaporation rate, and other inputs, the CNN model predicted water levels. These water levels, along with prior water quality data, were fed into the LSTM model, yielding CNN-LSTM having *R*^2^ values above 0.86 for training and over 0.79 for validation. In the Presa basin, Barzegar et al. (2020) employed a CNN-LSTM model to predict DO and chlorophyll a concentrations. The CNN model extracted periodic features from pH, conductivity, and other variables, which were then amplified by the LSTM. Training accuracy for DO and Chl-a reached correlation coefficients of 0.96 and 0.92, with validation at 0.97 and 0.87. The hybrid CNN-LSTM also outperformed other models. Sha et al. (2021) validated robust DO and TN predictions in the Xin'anjiang River using CNN-LSTM. Employing 12-day input data and employing complementary ensemble empirical mode decomposition with adaptive noise (CEEMDAN), the CNN-LSTM model demonstrated improved performance compared to standalone CNN and LSTM models. The preprocessing further enhanced predictions, improving Nash-Sutcliffe efficiency values for both DO and TN. In summary, these studies collectively showcase CNN-LSTM's capability in short-term water quality forecasting and highlight the impact of preprocessing on its performance.

Jang et al. (2021) utilized the LSTM-CNN model to forecast antibiotic-resistance gene (ARG) occurrences at Gwangalli Beach, Republic of Korea. Cumulative rainfall, tides, salinity, temperature, wind parameters, and humidity formed the input time-series data. LSTM initially extracted essential features, followed by CNN further refining these features to enhance ARG prediction. The LSTM-CNN model outperformed the sole LSTM, achieving *R*^2^ values between 0.20 and 0.67 for training and 0.15 to 0.55 for validation. The authors emphasized the LSTM-CNN's potential for predicting contaminants and water quality variables in aquatic settings. Furthermore, Yao et al. (2023) presented a data-driven deep learning model for runoff prediction, combining CNN-LSTM to improve prediction accuracy and versatility. The model uses meteorological, hydrological, and runoff data, reducing dimensionality with the maximum information coefficient. It employs CNN for long time series feature extraction, LSTM for long-term runoff prediction. Also, Liu et al. (2023) showed approach combining a two-stage feature selection of a hybrid deep learning model including CNN, LSTM, effectively captures the complex relationships in multivariate time series data from wastewater treatment plants (WWTPs). A two-stage feature selection process optimizes the feature subset to enhance prediction accuracy of predicting effluent total nitrogen in WWTPs.

These studies underscore the efficacy of coupling CNN and LSTM models for forecasting both water quality and contaminants in aquatic environments. Thus, the ensemble LSTM with CNN model offered a valuable approach for improving prediction accuracy with additional feature extraction of the data. Herein, CNN-LSTM specialized to deal with spatial data while LSTM-CNN particularized the time-series information.

## 4 LSTM with attention mechanism

### 4.1 LSTM-attention theory

Yet another method that can enhance feature extraction in a data series is including the attention mechanism in the LSTM model. The attention mechanism can improve the selection of input sequences and encode information in long-term memory (Li et al., 2019). Although LSTM addresses the long-term dependency problem, a vanishing gradient problem of network training may arise. This makes it difficult to capture distant past data, which may be valuable predictors. This implies that the performance of LSTM may not be satisfactory if important information is in the distant past from the time when the predictions are made. To avoid this issue, the LSTM model can focus on the relevant information using the attention mechanism rather than simply learning it in chronological order (Zheng et al., 2021b). Traditional LSTM, however, uses a simple concatenation of *h*^<^*^t^*^−1>^ and *x*^<^*^t^*^>^ as [*h*^<^*^t^*^−1>^, *x*^<^*^t^*^>^] (Fig. 1); the LSTM with attention applies attention weights to *x*^<^*^t^*^>^ before concatenation, and these weights are different for different gates. Attention weights are being determined during the training process. Through the back-propagation process, the attention weights are being optimized along with the weights of the LSTM.

### 4.2 Attention-LSTM application for water quality prediction

Recent studies have employed attention-LSTM in water quality modeling. Zhu et al. (2021) used a fusion model incorporating ResNet, BiLSTM, and multihead attention to predict DO concentrations in Lake Taihu. The attention mechanism extracted features from diverse environmental data, yielding superior results compared to LSTM without attention. Cao et al. (2021) applied attention-LSTM to simulate DO concentrations in ponds, achieving an RMSE of 0.380 mg/L. Cao et al. (2021) also explored attention's impact on the simpler GRU model, finding the attention-GRU outperformed both conventional GRU and attention-LSTM models. Jang et al. (2021) integrated input attention (IA) with LSTM for predicting ARG occurrence at a beach. IA-LSTM improved over conventional LSTM, though LSTM-CNN excelled in certain scenarios. IA-LSTM outperformed for multi-ARG simulations, while LSTM-CNN proved superior for single ARG predictions. This suggests attention mechanisms enhance LSTM's learning in intricate contexts.

Overall, the attention-LSTM model outperformed the conventional LSTM model in simulating water quality. The advantage of attention-LSTM models is that they can identify the relevance of information, which is not possible with the conventional LSTM model. However, attention-enhanced LSTM models are not always superior to other model combinations; therefore, the dataset-specific model structure must be carefully selected.

## 5 LSTM combination with transfer learning (TL)

Most water quality data are automatically measured by sensors at multiple monitoring stations in water-environment (Zhang et al., 2019). Numerous reasons (e.g., data entry errors, non-response, technical issues, and natural cases) may cause substantial missing values in raw water quality datasets (Ma et al., 2020a). These missing data significantly degrade water quality prediction using LSTM. Therefore, Transfer learning could be an alternative way to resolve limitations associated with missing water quality data.

### 5.1 LSTM-TL theory

Transfer learning (TL) is a methodology that transfers learned knowledge from one problem (source) to another but similar problem (target). This technique has been applied when a model is trained with insufficient or missing datasets. This can be achieved by transferring knowledge from the source domain (i.e., complete data) to the target domain (i.e., incomplete data); for example, TL can transfer knowledge from the complete data obtained at adjacent monitoring stations to missing data in the target domain (Chen et al., 2021). Transfer learning can be categorized into data pattern transfer (e.g., trend and statistical characteristics), model transfer (e.g., model structure and parameters), and task transfer (Pan and Yang, 2009). In LSTM applications, data pattern and model transfers have been used to overcome the missing-data problem (Chen et al., 2021; Zhou 2020). Data pattern transfer selects the source domain that has the greatest statistical similarity with the target domain, and both datasets are mixed and used to train the LSTM model. Model transfer means that the optimized structure (multiple output and number of hidden layers) and parameters (learning rate, weight vector, and bias vector) from the source domain can be transferred to the LSTM model in the target domain (Zhou, 2020).

### 5.2 LSTM-TL application for water quality predictions

**Fig. 3:**
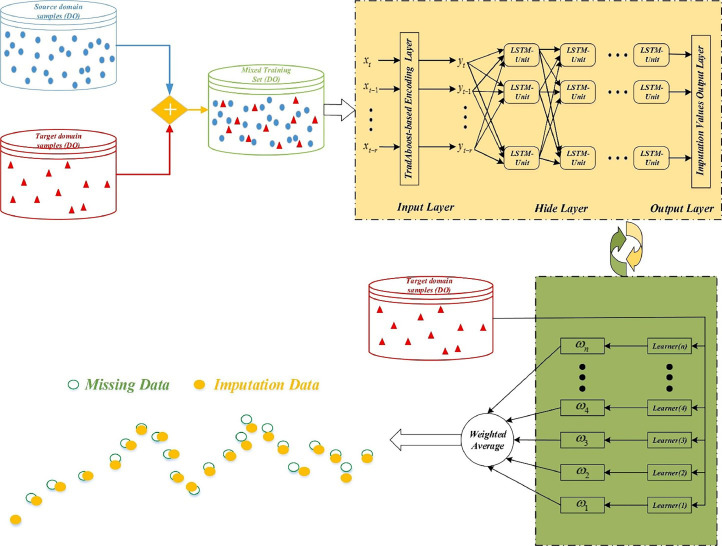
Proposed large-scale consecutive missing-data imputation transfer algorithm based on TrAdaBoost-LSTM (From Chen et al., 2021).

Zhou (2020) employed data pattern and model transfers to counteract missing data effects. Comparing general LSTM and TL-LSTM, they assessed various missing-data rates and forecast scenarios. TL-LSTM's effectiveness was demonstrated in both data missing rates and forecast lead-time situations. For instance, at a 10-day lead and 90 % data missing rate, TL-LSTM improved RMSE and NSE by 24.7 % and 23.3 % respectively. Chen et al. (2021) used TL-LSTM with TrAdaBoost to impute missing DO concentration data, achieving 15–25 % better imputation performance than other methods (Fig. 3). Similarly, Tian et al. (2019) optimized LSTM for Chl-a concentration prediction using TL, outperforming parameter norm penalties and dropout methods over a 3-month period. Thus, these studies highlight the potential of transfer learning to enhance water quality modeling and prediction accuracy under varying conditions.

## 6 Influence of data transformation and static inputs on the model performance

**Fig. 4:**
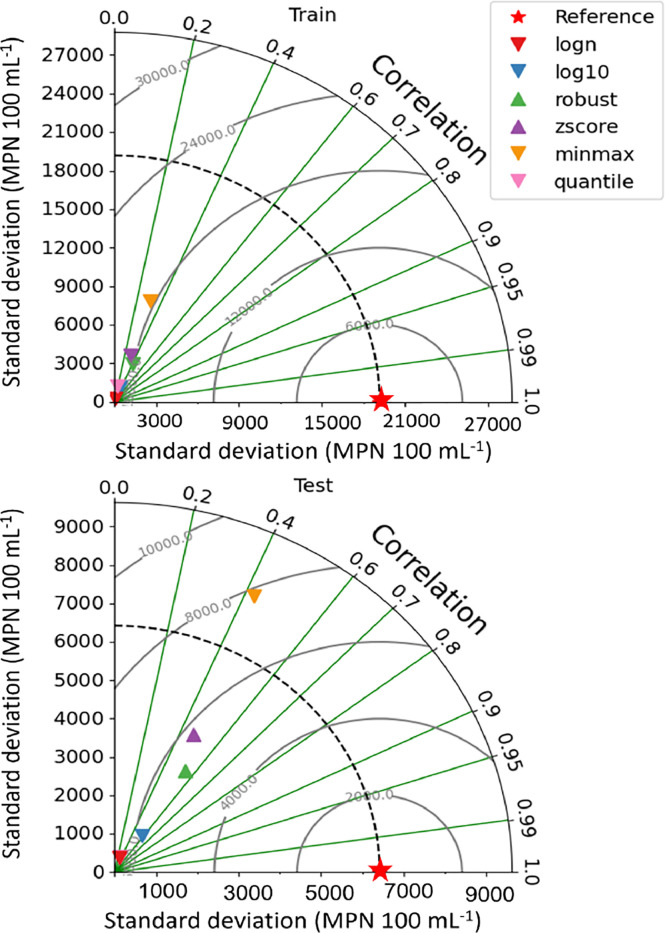
Influence of data transformation on the model performance (Abbas et al., 2022) in terms of correlation, standard deviation; the closer to the reference (red star), the better model performance.

Data transformation plays an important role in determining the performance of LSTM for water quality prediction. The selection of data transformation methods can substantially influence how good the LSTM captures patterns, handles temporal dependencies of water quality. Abbas et al. (2022) observed the influence of data transformations on the model performance. The results of LSTM with the logarithmic input transformation were closer to the observations than those of the min–max transformation, yielding an NSE of 0.57 (Fig. 4). A negative Percent Bias value was obtained after logarithmic transformation. This indicated that the simulated *Escherichia coli* from the logarithmic transformation was underestimated, whereas the result of the min–max transformation was overestimated. This behavior may be attributed to the higher sensitivity of min–max scaling to outliers (Chuang et al., 2010).

Static inputs can have a significant influence on the performance of LSTM models in water quality prediction. These static inputs do not change over time and can provide additional information to the LSTM model. Incorporating static inputs into LSTM models can improve their ability to capture complex relationships and increase prediction accuracy. Efforts to integrate static attributes into LSTM have been explored. Lees et al. (2021a) introduced an entity-aware LSTM (EA-LSTM) for hydrological pattern learning based on static catchment features, yet their study indicated a performance decline compared to basic LSTM. Abbas et al. (2023) incorporated sub-basin characteristics as static data alongside continuous input data for LSTM. To address static data's lack of temporal information, they initialized LSTM's hidden and cell states with static values, assuming this strategy would provide predictive insights.

To investigate the importance of static input data, they compared the performance of the model with an LSTM model without static data. The results also indicated that, without using static variables, the performance of LSTM was worse. Another example, Frame et al. (2021) utilized LSTM with both hydrological static and dynamic data to improve the accuracy of predicting stream flow. They noted that altering the routing mechanism could preserve temporal details from the land surface processes, while adjustments to the evapotranspiration choices might maintain information related to mass bias. These imply that the use of static physical features of sub-basins to initialize hidden and cell states provides important contextual information to the LSTM. This information regarding the physical features of a sub-basin improves the LSTM prediction performance at the validation site. The simultaneous learning of between time series and static physical features leverages the ability of LSTM training and evade the unrealistic parametrization of applying traditional deterministic modeling (Kratzert et al., 2019a,b). In addition, using the static input feature of LSTM attribute to direct application for the cases that have similar regional and physical information (Drost et al., 2021).

## 7 Summary: LSTM-based approaches for water quality simulation

We reviewed various LSTM-based models used in water quality analysis and their key features, along with potential challenges and concerns associated with each model. Table 1 presents concise summary of each model. CEEDAN-LSTM preprocesses the data to distinguish and remove noise from periodic elements, then applies this processed data as input to an LSTM model. Care should be taken when interpreting how CEEMDAN components and LSTM states interact, particularly in the context of water quality analysis. VMD-LSTM effectively manages multi-scale and non-linear temporal patterns in water quality data. There is a potential risk of overfitting due to incomplete regularization and validation. CEEDAN-VMD-LSTM employs a dual-stage decomposition method to optimize information attributes. This model demands careful focus on hyperparameter adjustment, data preparation, and model interpretation. Sequenced-LSTM employs multiple LSTMs to capture autoregressive elements from the original water quality data. There are possible challenges related to vanishing and exploding gradients should be considered. Wavelet-LSTM breaks down signals into various distinct patterns through the application of wavelet functions. It is crucial to carefully select the wavelet function and effectively manage boundary effects and sensitivity to noise. CNN-LSTM adopts convolutional layers to extract features from the data. This approach may introduce increased model complexities, necessitating meticulous hyperparameter tuning. LSTM-CNN utilizes CNN as a post-processing step within the model. This may lead to heightened model complexities and requires thorough attention to hyperparameter tuning. Attention-LSTM concentrates on specific segments of input sequences during processing via attention mechanisms. There is a potential for overfitting and heightened computational demands that should be considered. TL-LSTM helps leverage information from one domain to improve performance in another but challenging when dealing with mismatched domains.

**Table 1:** Features and a cautionary note of LSTM-based models.

Model	Features	A cautionary note
CEEDAN-LSTM	A preprocessing process for extracting to separate the separate the noise from periodic components of the environmental data and used as inputs of the LSTM model.	Interpreting interactions between CEEMDAN components and LSTM states (e.g., water quality)
VMD-LSTM	A preprocessing process to effectively handle and model multi-scale and non-linear temporal patterns within water quality-related data	Overfitting problem caused by incomplete regularization and validation
CEEMDAN-VMD-LSTM	A two-stage decomposition technology maximizes the information characteristics	High level of care and attention to detail in hyperparameter tuning, data preparation, and model interpretation
Sequenced-LSTM	Usage of multiple LSTMs to extracts the autoregressive component of the original water quality data	Vanishing and Exploding Gradients
Wavelet-LSTM	A method to decompose a signal into multiple and special pattern	The choice of wavelet function and handling boundary effects and noise sensitivity.
CNN-LSTM	Usage of CNN for feature extraction from the data	Higher complexities and hyperparameter tuning
LSTM-CNN	Usage of CNN as a post-processing process.	Higher complexities and hyperparameter tuning
Attention-LSTM	Focusing on specific parts of input sequences during processing	Overfitting and computation demands
TL-LSTM	Helping leverage information from one domain to improve performance in another.	Mismatched domains

Each model has its unique strengths and challenges, and careful consideration of these features and cautions is essential when choosing the appropriate model for a specific water quality simulation.

## 8 Outlook: challenges and opportunities

### 8.1 Integrating multi-sources, extension, and generalization

Water quality is influenced by various sources including land use, climate, and agricultural activities. Incorporating data from multi-sources (e.g., static covariates or diverse time-varying variables) into LSTM models is one of major challenges. Therefore, it is very crucial to apply robust feature selection and dimensionality reduction methods to handle multi-sources data.

The LSTM approach can generate predictions using only forward data information, whereas a bi-directional LSTM (Bi-LSTM) method considers both forward and backward neural network information to make predictions. The Bi-LSTM effectively captures past and future information independently at specific time steps through its dual LSTM. Thus, by incorporating both positive and negative time series data rules, the extension of LSTM model, Bi-LSTM, may achieve higher prediction accuracy compared to LSTM (Tan et al., 2023).

LSTM models trained with specific water quality station might struggle to generalize to another stations with distinct characteristics even in same watershed. Here, Transferability and accurate prediction of LSTM across multiple stations in same or different watersheds remain challenging. Effectively incorporating transfer learning by pre-training on similar water quality station could be one of solutions to the resolve the issue. Leveraging domain adaptation techniques can help the LSTM generalize successfully (Deng et al., 2022)

### 8.2 Model interpretability and explainability

Interest in explaining the mechanism of deep learning models is increasing (Floridi, 2021). This is more the case when making high-stake decisions based on the results of LSTM-based modeling (Lipton, 2018). Therefore, it is not only important to make good predictions using LSTM models but also to explain them. The methods used to explain the decision-making process of LSTM can be categorized into two types: 1) model-agnostic interpretation and 2) model-dependent interpretation (Molnar et al., 2020a). Model-agnostic interpretation methods consider the LSTM as a black box and explain its behavior by analyzing its output against perturbed inputs (Molnar et al., 2020b). However, these methods have also received criticism due to inconsistencies and instability in their results (Rudin, 2019). On the other hand, model-dependent interpretation methods rely on the internal state of LSTM, including the weights, cell state, and hidden state of LSTM. These methods employ post-hoc approaches, such as integrating the gradients or using simple linear regression (SLR) on the internal elements of LSTM (i.e., cell and hidden states) to explain the decision-making process in LSTM (Lees et al., 2021b). The use of SLR to explain the behavior of deep learning models is common in natural language processing (Hewitt and Liang, 2019). Recently, Lees et al. (2021b) conducted a study in which the cell state of an LSTM was interpreted with the help of SLR. The objective of this study was to determine whether LSTM can learn a hydrologically realistic phenomenon during training. They found that, during the training of LSTM to model the rainfall-runoff process, the internal elements of LSTM learn an intermediate phenomenon (e.g., changes in water storage in the soil). The study showed that the cell state of LSTM after the application of SLR can represent soil moisture. The use of SLR to interpret the internal variables of LSTM can be extended to water quality modeling. LSTM has been successfully used to model micropollutants and harmful algal blooms in surface waters (Yun et al., 2021; Zheng et al., 2021a). However, these models only provide target prediction at a specific site and do not indicate whether the LSTM has learned a representative related-phenomenon. The use of SLR along with LSTM, as shown by Lees et al. (2021b), can be adopted for this purpose.

Another approach used for making LSTM results more tenable is to force the model to follow physical laws by encoding these natural laws inside the LSTM. One such example is the mass conservation LSTM (MC-LSTM), in which the mass conservation principle is encoded in the equations of the LSTM (Hoedt et al., 2021). In the MC-LSTM, the mass of the target input inside the cell state is conserved over time. This is performed by combining the gating mechanism of LSTM and the redistribution of input in the cell state of LSTM. The authors argue that MC-LSTM can be extended to conserve variables such as energy, momentum, and count. Because energy and mass conservation laws are fundamental parts of water quality models (Neitsch et al., 2011), the use of MC-LSTM can make physically consistent and realistic water quality predictions.

### 8.3 Handling availability and data quality

A major challenge in the applicability of LSTM for water quality modeling is the scarcity of observation data. Monitoring and analyzing of water quality parameters is time consuming and costly (Baek et al., 2021). However, the development of data-driven models with high prediction performance requires a large amount of data (Goodfellow et al., 2016). There have been attempts to overcome this shortcoming by incorporating domain knowledge into the LSTM along with input data. This mitigates the potential breaches of physical laws that might arise due to limited input data (Zhang et al., 2020). The domain knowledge is given to the model in the form of constraints and boosts on the loss function which makes the solution within a feasible and realistic solution space (Jia et al., 2018; Karpatne et al., 2017; Raissi, 2018; Raissi et al., 2019).

In water quality modeling, even when large amounts of data are available, they are usually not monitored at a uniform time step, which is an essential requirement for time-series modeling using LSTM Chollet, 2018). Usually, water quality is monitored at irregular time steps or during certain seasons or special time intervals, such as during rainfall events (Jang et al., 2021). The LSTM enhances with ordinary differential equation (ODE-LSTM) has been specifically designed to model irregularly sampled time-series data (Rubanova et al., 2019). In the ODE-LSTM, the states of LSTM are considered as continuous time representations and modeled by solving the ODE at every time step using a numerical ODE solver (Lechner and Hasani, 2020). This means that, instead of using standard LSTM equations (Eqs. (1)–((6) to calculate its states, ODE-LSTM solves the initial value problem between two observations and calculates the LSTM state (Eq. (6)) (Rubanova et al., 2019). This feature of ODE-LSTM makes it a natural tool for modeling irregularly sampled water quality data.

### 8.4 Efficient optimization

Optimizing LSTM models helps in finding the best combination of hyperparameters that can exploit the unique capabilities of LSTMs to their fullest extent, leading to more accurate predictions, reduced training time, and ultimately better performance on various tasks involving sequential data. In particular, employing machine learning techniques for optimizing LSTM models enhances the efficiency, effectiveness, and performance of these models, making them more suitable for real-world applications involving sequential data. Dheda et al. (2022) introduced Genetic Algorithm (GA) optimization with LSTM to enhance DO prediction performance of Burnnett River and Baffle River in Australia. In GA-LSTM model, throughout the process of optimization, the genetic operators were utilized to explore the potential solutions within the search space. This led to the formation of a population consisting of conceivable solutions, represented as chromosomes encoded using binary bits. These chromosomes conveyed information about the optimal hyperparameter values, specifically indicating the quantity of LSTM units and the size of the time window. The LSTM models that underwent optimization using the GA demonstrated superior performance compared to their initial versions. This improvement in model performance was achieved by fine-tuning hyperparameters through the GA optimization process. The most significant outcome of GA optimization was the reduction in both the overall computational time required and the count of parameters that could be trained in the models.

### 8.5 Fusion with statistical and process-based model

The combination of LSTM and statistical modeling explores the synergistic integration of deep learning and statistical techniques in inland water environment. This approach aims to leverage LSTM's ability to capture complex temporal dependencies in data alongside the strengths of statistical modeling, which provides interpretability, robustness, and probabilistic insights. This fusion helps accomplish time series prediction, anomaly detection, and uncertainty quantification for water environment analysis. Thus, unique perspective on the fusion of LSTM and statistical inference opens up the potential for improvement of data analysis and predictive modeling (Fang et al., 2022).

Theory-Guided machine learning is the other promising approach by combining process-based and LSTM models. Frame et al. (2021) proposed LSTM postprocessors providing significant benefit to enhance the performance of United States National Water Model (NWM). Employing LSTM for post-processing offers the potential to enhance the interpretability. Their findings revealed a strong correlation between the representation of hydrologic characteristics in the LSTM model and NWM. This suggests that the functions learned by the LSTM, which map inputs to streamflow, exhibit substantial similarity. Furthermore, even though articulating the intricate LSTM behavior concisely through compact formulas like partial differential equations might be challenging due to the numerous trained model parameters, LSTM model can recognize their structural resemblances to process-based models such as the NWM.

In conclusion, the future research of LSTM in the water environment depends on tackling these bottlenecks through interdisciplinary and innovative collaboration. Tackling these challenges will lead to more accurate, robust, and practical LSTM-based water quality prediction models, contributing to effective water resource management and environmental protection.

## 9 Conclusion

The major findings from our review are as follows:

• LSTM models demonstrate robust performance for water quality prediction compared with other machine learning models. They can extract the dynamic features of water quality, store the most valuable time-frequency information, and achieve accurate predictions.
• Combining LSTM and CNN improves predictions by both letting CNN to process LSTM output and letting LSTM to process CNN output.
• Being an efficient prediction tool, LSTM can be further enhanced by combining it with attention mechanism and transfer learning approach.
• Better interpretation of results and efficient use of relevant non-time series-type information present both challenges and opportunities for the development of the LSTM-based data analysis and prediction tools in water quality area.
• Using the watershed static information and the data transformation method can substantially enhance the LSTM performance.

## Declaration of Competing Interest

The authors declare that they have no known competing financial interests or personal relationships that could have appeared to influence the work reported in this paper.

## Data Availability

Data will be made available on request.
